# Hypoxia, blood flow and metabolism in squamous-cell carcinoma of the head and neck: correlations between multiple immunohistochemical parameters and PET

**DOI:** 10.1186/1471-2407-14-876

**Published:** 2014-11-24

**Authors:** Tove J Grönroos, Kaisa Lehtiö, Karl-Ove Söderström, Pauliina Kronqvist, Jukka Laine, Olli Eskola, Tapio Viljanen, Reidar Grénman, Olof Solin, Heikki Minn

**Affiliations:** Turku PET Centre, Medicity Research Laboratory, University of Turku, Tykistökatu 6 A, FI-20520 Turku, Finland; Department of Oncology and Radiotherapy, Turku University Central Hospital, Turku, Finland; Department of Pathology, University of Turku, Turku, Finland; Department Otorhinolaryngology and Head and Neck Surgery, Turku University Central Hospital, Turku, Finland

**Keywords:** [^18^F]FETNIM, [^18^F]FDG, Blood flow, Hypoxia, Head and neck cancer, Immunohistochemistry

## Abstract

**Background:**

The relationship between the uptake of [^18^F]fluoroerythronitroimidazole ([^18^F]FETNIM), blood flow ([^15^O]H_2_O) and 2-[^18^F]fluoro-2-deoxyglucose ([^18^F]FDG) and immunohistochemically determined biomarkers was evaluated in squamous-cell carcinomas of the head and neck (HNSCC).

**Methods:**

[^18^F]FETNIM and [^18^F]FDG PET were performed on separate days on 15 untreated patients with HNSCC. Hypoxia imaging with [^18^F]FETNIM was coupled with measurement of tumor blood flow using [^15^O]H_2_O. Uptake of [^18^F]FETNIM was measured as tumor-to-plasma ratio (T/P) and fractional hypoxic volume (FHV), and that of [^18^F]FDG as standardized uptake value (SUV) and the metabolically active tumor volume (TV). Tumor biopsies were cut and stained for GLUT-1, Ki-67, p53, CD68, HIF-1α, VEGF_sc-152_, CD31 and apoptosis. The expression of biomarkers was correlated to PET findings and patient outcome.

**Results:**

None of the PET parameters depicting hypoxia and metabolism correlated with the expression of the biomarkers on a continuous scale. When PET parameters were divided into two groups according to median values, a significant association was detected between [^18^F]FDG SUV and p53 expression (p =0.029) using median SUV as the cut-off. There was a significant association between tumor volume and the amount of apoptotic cells (p =0.029). The intensity of VEGF stained cells was associated with [^18^F]FDG SUV (p =0.036). Patient outcome was associated with tumor macrophage content (p =0.050), but not with the other biomarkers. HIF-1α correlated with GLUT-1 (r_s_ =0.553, p =0.040) and Ki-67 with HIF-1α (r_s_ =506, p =0.065). p53 correlated inversely with GLUT-1 (r_s_ = −618, p =0.019) and apoptosis with Ki-67 (r_s_ = −638, p =0.014).

**Conclusions:**

A high uptake of [^18^F]FDG expressed as SUV is linked to an aggressive HNSCC phenotype: the rate of apoptosis is low and the expressions of p53 and VEGF are high. None of the studied biomarkers correlated with perfusion and hypoxia as evaluated with [^15^O]H_2_O-PET and [^18^F]FETNIM-PET. Increased tumor metabolism evaluated with PET may thus signify an aggressive phenotype, which should be taken into account in the management of HNSCC.

## Background

The microenvironment of cancer tissues is very different from that of healthy tissue. There is uncontrolled formation of new blood vessels in tumors and this results in chaotic and heterogeneous tumor vascularization. Consequently, tumor blood flow is variable causing irregular metabolic gradients, particularly gradients in the oxygen and glucose concentrations [1]. Blood flow data on human tumors in situ are scarce, but the few existing studies indicate that the blood flow varies significantly depending upon tumor type, size and site of growth. A considerable heterogeneity of flow rates can even be observed in tumors with identical histological classifications [2].

Many human malignancies exhibit hypoxic tissue areas that are heterogeneously distributed within the tumor mass; these may be located even adjacent to well-perfused areas [1]. The initial molecular response to hypoxia is mediated through the hypoxia-inducible transcription factor-1α (HIF-1 α). In the absence of oxygen, HIF-1α binds to hypoxia-response elements (HREs), thereby activating the expression of numerous hypoxia-response genes such as those involved in angiogenesis, glycolysis and oxygen delivery. In general, one could say that the cellular response to hypoxia is intended to prevent cell death and indeed an increased level of intracellular HIF-1α has been associated with a poor prognosis and resistance to therapy in cancer [3]. In addition to the fact that hypoxia upregulates glycolysis, classical biochemical studies have shown high rates of glycolysis in cancer cells, independent of the presence of oxygen (Warburg’s effect) [4]. The molecular mechanisms leading to the upregulation of glycolysis in tumors are still not well understood [5]. In addition to elevated glycolysis, tumors often show an increased expression of glucose transporters and/or hexokinase activity in comparison to normal tissues.

A high metabolic rate indicated by high [^18^F]FDG uptake seems to be a predictor of poor outcome for many tumor types [6]. This predictive capacity might be a consequence of the fact that the elevated glycolysis encountered in tumors is related to several biological factors associated with poor prognosis, including hypoxia [7], accelerated cell proliferation [8], inflammation [9] and reduced apoptosis [10].

Hypoxic cells are approximately three-fold more resistant to radiation therapy than well-oxygenated cells. Several ^18^F-labelled 2-nitroimidazole compounds have been evaluated for their usefulness as hypoxia tracers with PET [11]. So far, [^18^F]FMISO is the only one of these tracers that has widely become used in the clinic. Since hypoxia is known to increase glycolysis [^18^F]FDG has also been proposed as a potential tracer for imaging of hypoxia. Although increased uptake of [^18^F]FDG might indicate the presence of some degree of hypoxia [7] [^18^F]FDG has not proved to function as a surrogate tracer for hypoxia [12].

We have previously described the pharmacokinetic properties of [^18^F]FETNIM as a hypoxia tracer in experimental tumors [13, 14] and in patients with squamous-cell carcinoma of the head and neck (HNSCC) [12, 15, 16]. [^18^F]FETNIM PET studies in patients with HNSCC were combined with blood flow measurements utilizing [^15^O]H_2_O and [^18^F]FDG. Although [^18^F]FETNIM showed a lower and more favorable background signal than [^18^F]FMISO [14], the high hydrophilicity of [^18^F]FETNIM led to early tumor uptake, which was largely perfusion dependent up to 90 min post injection [15]. Generally, a 5- to 30-fold greater blood flow was seen in tumor than in muscle. A high uptake of [^18^F]FETNIM prior to radiation therapy was associated with a trend towards poor overall survival, whereas [^18^F]FDG SUV (p =0.028) and blood flow (p =0.018) were clearly associated with poor patient survival [12].

To gain a wider knowledge of the physiological and pathological changes behind the uptake of tracers believed to describe glucose metabolism, hypoxia and blood flow, we compared the expression of multiple biochemical biomarkers with the uptake of [^18^F]FETNIM, [^18^F]FDG and [^15^O]H_2_O as well as the patient outcome in patients with HNSCC. Immunohistochemistry and in situ methods were used to determine the expression of the glucose transporter (GLUT-1), hypoxia-inducible transcription factor-1 (HIF-1α), vascular endothelial growth factor (VEGF), microvessel density (CD31), macrophages (CD68), proliferation (Ki-67), p53 expression and apoptosis (Tunel) in biopsy samples from patients who had earlier participated in a multitracer PET study [12]. All of the selected biomarkers are endogenous molecules that might be involved in, or influence, the underlying biological pathways responsible for the uptake of the investigated tracers. In addition, the expression of these selected biomarkers was correlated with patient outcome.

## Methods

### Patients and tissues

The PET study protocol and the consent form were approved by the ethics committee of the Turku University Central Hospital and permission to use [^18^F]FETNIM in patient studies was granted by the Finnish National Agency for Medicines. All patients provided written informed consent before entering the study. All PET studies were performed before any oncologic treatment was given. The use of tumor samples for molecular analysis was approved by the National Authority for Medicolegal Affairs.

Fifteen patients with newly diagnosed head and neck carcinoma (tumor category T1-T4) and with a variety of primary tumor site presentations participated in the study (Table 1). All patients were part of an earlier study on 21 head and neck cancer patients imaged with [^18^F]FDG, [^18^F]FETNIM and [^15^O]H_2_O [12]. Only patients with histologically confirmed squamous cell carcinoma and representative biopsy material were included in this study. Excisional biopsies were taken from the patients during panendoscopy by a specialist in otolaryngology. The otolaryngologist who obtained samples was blinded to the imaging results and not involved in the study at any other level. The maximum time elapsing between extraction of tumor biopsies and the performed PET scans was 30 days (median 19, range 7–30). All patients received either definitive or preoperative external beam radiotherapy (RT) at doses ranging from 60 to 70 Gy (Table 1). Two patients (Patients 12 and 14) received concomitant chemotherapy consisting of cisplatin and fluorouracil. Paraffin-embedded tissue blocks of formalin-fixed samples were processed for histological study and immunohistochemical analysis. After treatment, the patients were followed until December 2005 or death. The median follow-up time after the diagnosis of cancer was 32 months (range 26–35).

**Table 1:** **Characteristics of patients with HNSCC**

Patient no.	Tumor site	TNM at diagnosis	Tumor stage	Differentiation	Type and doses of RT (Gy)	Survival in months
1	supraglottic larynx	T1N0M0	I	well	definitive/68.7	28*
2	supraglottic larynx	T2N0M0	II	moderate	definitive/70.0	52*
3	oral cavity	T3N2M0	IV	poor	preoperative/63.4	4*
4	oral cavity	T4N1M0	IV	moderate	preoperative/62.3	10*
5	hypopharynx	T1N3M0	IV	poor	preoperative/62.3	32*
6	oral cavity	T4N2M0	IV	moderate	preoperative/63.0	17*
7	glottic larynx	T2N0M0	II	moderate	definitive/70.0	64
8	hypopharynx	T4N1M0	IV	poor	preoperative/44.0	7*
9	oropharynx	T3N2M0	IV	moderate	preoperative/60.0	12*
10	oral cavity	T2N0M0	II	well	preoperative/64.6	63
11	nasopharynx	T3N0M0	III	poor	preoperative/63.6	70
12	nasopharynx	T3N2M0	III	poor	definitive/68.4	59
13	oropharynx	T1N2M0	IV	moderate	preoperative/62.4	59
14	oropharynx	T4N2M0	IV	moderate	preoperative/61.5	6*
15	oral cavity	T2N0M0	II	poor	preoperative/64.0	58

*Patients are no longer alive.

RT = radiotherapy.

### PET imaging and image analysis

The syntheses of [^18^F]FDG, [^18^F]FETNIM and [^15^O]H_2_O have been described previously [13, 15]. The PET studies were performed with a GE Advance PET scanner (General Electric Medical Systems, Milwaukee, WI, USA) operated in 2D mode. PET acquisition and image analysis have been described previously in detail [12].

In short, [^18^F]FDG was injected intravenously as a 15 second bolus (median dose 371 MBq, range 355–385 MBq) and a static emission scan consisting of three 5 min frames was acquired 45–60 min after the injection followed by a 10 min transmission scan. Dynamic [^18^F]FETNIM (median dose 368 MBq, range 289–385 MBq) studies were performed sequentially i.e. after the blood flow measurements using [^15^O]H_2_O (median dose 1150 MBq, range 821–1800 MBq).

[^18^F]FDG accumulation was measured as a standardized uptake value (SUV). Regions of interest (ROIs) were drawn into the time frame between 55 and 60 min after the injection. Tumor ROIs were defined by an isodensity contour tool using SUV of 4 as the threshold value. When necessary, parallel reading of corresponding axial computer tomography (CT) scans and/or clinical information was available in defining the tumor area. Volumes of these ROIs in all planes where the tumor was visible were summed to obtain the metabolically active volume of the tumor, which is known to correlate strongly with the volume determined by CT [17]. The plane with the highest 3 × 3 pixel (7.04 × 7.04 mm) maximum SUV, and two adjacent planes were carefully matched with the corresponding planes on the flow and [^18^F]FETNIM images. From [^18^F]FETNIM images, tumor-to-plasma ratios (T/P ratio) were calculated using data acquired 90 – 120 min after injection of tracer. The fractional hypoxic volume (FHV) of the tumor was determined in the following way. Large ROIs were first drawn in three adjacent planes in brain, muscle and lung tissues of 3 patients each. Secondly, tissue-to-plasma radioactivity ratios of all individual pixels (n =10968) in all these planes were pooled. Thirdly, the threshold for hypoxia was set at three standard deviations above the mean of these normal tissue-to-plasma activity ratios (=0.93). Finally, the percentage of pixels in whole tumor ROI above this ratio of 0.93 was calculated to obtain the FHV. Blood flow was measured with [^15^O]H_2_O utilizing the autoradiographic method using a 250-sec integration time and an arterial input curve. The process has been described in detail previously [15]. PET scans were analyzed by KL under the supervision of HM. In case of discrepancies a consensus reading was performed. Quantitative image analysis was done by KL and VO.

### Histology and immunohistochemistry

The necrotic tumor volume, degree of inflammation and estimates of mitoses, macrophages and apoptosis were obtained from hematoxylin-eosin stained tumor sections by conventional histological evaluation.

Immunohistochemistry was performed on 4-μm thick tissue sections. After deparaffination and rehydration, endogenous peroxidase activity was blocked for 30 minutes in an aqueous solution containing 0.3% hydrogen peroxide. Antigen retrieval was carried out in a microwave oven. The sections were then incubated with the primary antibody for 25 minutes at room temperature (RT). Visualization of primary antibodies was done with Vectastain ABC reagent and diaminobenzidine substrate kit (Vector Laboratories, Burlingame, CO), which is based on an indirect streptavidin-biotin method. The slides were later counterstained with hematoxylin. The antibodies and dilutions used were as follows: GLUT-1 (DAKO, Carpinteria, CA; dilution 1:200), VEGF_sc-152_ (Santa Cruz Biotechnology, Santa Cruz, CA; dilution 1:200) and HIF-1α (BD Transduction Laboratories, San Jose, CA; dilution 1:100). The staining for Ki-67 (DAKO; dilution 1:100), p53 (DAKO; dilution 1:300), CD31 (BioGenex, San Ramon, CA; dilution 1:2) and CD68 (DAKO; dilution 1:100) was done using the TechMate 500 immunostainer and a peroxidase/diaminobenzidine multilink detection kit (DAKO). Appropriate positive controls were used throughout the studies.

### In situ detection of apoptotic cells (TUNEL)

In situ detection of apoptotic cells in paraffin wax sections was performed as described earlier [18] with slight modifications. Briefly, endogenous peroxidase activity was blocked and DNA 3`-end-labeling was performed with terminal transferase buffer (Promega, Madison, WI). The reaction was allowed to continue for 1 hr at 37°C in a humidified chamber. Slides were then incubated with blocking buffer containing 2% blocking reagent and 0.05% sodium azide (Boehringer) for 30 min. Antidigoxigenin antibody, conjugated to alkaline phosphatase (1:2000, Boehringer), in 2% blocking buffer was added and incubated for 2 hr. The slides were treated with alkaline phosphatase buffer for 10 min. Thereafter, 337 mg/ml nitroblue tetrazolium salt (Boehringer) and 175 mg/ml 5-bromo-4-chloro-3-indoylphosphate (Boehringer) were added in fresh alkaline phosphatase buffer, and the reaction was terminated 3 hr and 45 min later by addition of 1 mM EDTA and 10 mM Tris–HCl, pH 8.0. Finally, slides were mounted with Gurr Aquamount (BDH Chemicals, Poole, UK). For controls, terminal transferase, dig-ddUTP, or antidigoxigenin antibody were omitted from the reaction.

### Data analysis

An experienced pathologist examined the hematoxylin-eosin stained samples and was blind to all other biomarkers and PET parameters. The percentage of necrotic tumor volume was estimated and the degree of inflammation and the amount of mitoses, macrophages and apoptoses was semiquantitatively scored as none, slight, moderate or severe.

All immunohistochemical analyses were conducted by two independent observers who were unaware of the PET data. All sections were first evaluated with a ×20 objective as to provide an estimation of cells showing staining in the whole sample. The most representative tumor area was identified and a quantitative assessment of the percentage of cells showing nuclear staining in the ×40 objective in three separate optical fields in a total of 300 carcinoma cells was calculated from sections stained for p53 expression. The percentage of cells showing staining in the cytoplasm was calculated for CD68 in a similar manner. For HIF-1, similar calculations were done in hot spot areas showing nuclear staining. In this study, we counted the Ki-67 expression from a total of 300 carcinoma cells in invasive regions only. Tumor cells were considered positive for GLUT-1 expression whenever an even slight netlike membrane staining was present regardless of the degree of the cytoplasmic staining pattern. Again, the percentage of positive cells from a total of 300 carcinoma cells was calculated. Tumor cells showing VEGF_sc-152_ staining in the cytoplasm was scored according to the intensity of the staining as weak, moderate or intense depending on the area within the tumor that revealed the most intense staining (hot spot). For further analysis tumors were divided into two groups that represented tumors with weak staining (n =6) and intense (moderate or strong) staining (n =9). Within the CD31 stained slides, the microvessel hot spot area was identified and microvessels were counted with ×40 magnification and expressed as a percentage of vessels per square millimetre.

Apoptotic cells detected with Tunel were counted from tumor sections stained with the antidigoxigenin antibody. The presence of a distinct intensely dark color reaction within tumor cells was regarded as representing apoptotic DNA fragmentation. The results are expressed as number of positive cells per millimetre squared when a ×10 objective lens was used. In situ detection of free DNA 3’-ends is a well-established method for the detection of apoptotic cellular changes, and this was validated by simultaneous electrophoretic DNA analysis in pancreatic tissue [18].

### Statistical analyses

Statistical analyses were performed with SAS System software (Service Pack 2), version 9.1.3 (SAS Institute, Cary, NC, USA). Nonparametric tests were used throughout since the assumption of normality was violated in some parameters. Spearman’s correlation coefficient (r_s_) was used to correlate PET parameters with histological findings. Due to the limited sample size, no adjustment for simultaneous testing of multiple variables was performed. The Wilcoxon rank sum test was used to compare histological findings in PET parameter groups (dichotomized using the median as the cut point) and clinical outcome. The limit for statistical significance was set at p <0.05.

## Results

### Relationship between PET findings and immunohistochemistry

The immunostaining displayed a heterogeneous expression pattern. The median (range) of positive stained cells analyzed from samples was 40% (17 – 87%) for Ki-67, 25% (0 – 60%) for GLUT-1, 70% (2 – 95%) for p53, 16% (0 – 68%) for HIF-1α, 27% (5 - 44%) for CD68, 3% (0.4 – 14.9%) for CD31 and 10% (0.3 – 20%) for apoptosis as detected with the Tunel method.

Individual PET findings for the 15 patients are presented in Table 2 and biomarker findings in Table 3. Spearman’s correlation coefficients (r_s_) and p-values were calculated from PET for the relationship between [^18^F]FDG, [^18^F]FETNIM or [^15^O]H_2_O, and the expression of biomarkers (Table 4). When the tracer uptake indices were treated as continuous variables no correlation could be detected between the PET data and any of the endogenous biomarkers, although the expression of p53 showed a trend toward a correlation with [^18^F]FDG SUV (r_s_ =0.470, p =0.078), as shown in Table 4.

**Table 2:** **Quantitative analyses of PET findings using three tracers in patients with HNSCC**

	[ ^18^F]FDG		[ ^18^F]FETNIM		[ ^15^O]H _2_O
Patient no.	SUV ^a^	Tumor volume ^b^ (cm ^3^)	FHV ^c^ (%)	T/P ratio ^d^	Blood flow (ml/100 mg/min)
1	18.5	8.3	9.5	0.91	35.3
2	14.9	3.4	10.5	0.72	29.7
3	19.0	34.1	61.6	1.41	44.4
4	8.4	9.9	61.4	1.98	63.1
5	17.3	401.6	39.2	1.49	44.8
6	11.7	53.6	54.2	1.24	23.7
7	7.6	1.7	17.8	0.95	12.4
8	18.3	141.1	55.7	1.34	29.6
9	13.0	22.2	48.0	1.07	n.d.
10	7.8	5.5	50.5	1.11	26.2
11	28.8	23.6	19.7	1.00	19.7
12	9.6	12.6	34.0	1.02	37.9
13	7.2	5.0	10.1	0.82	24.3
14	19.6	52.6	63.2	1.37	41.0
15	5.3	1.4	62.3	1.10	31.0
**Mean ± SD**	**13.8 ± 6.5**	**51.8 ± 103.2**	**39.8 ± 21.1**	**1.17 ± 0.32**	**33.1 ± 12.7**
**Median (range)**	**13.0 (5.3 – 28.8)**	**12.6 (1.4 – 401.6)**	**48.0 (9.5 – 63.2)**	**1.10 (0.72 – 1.98)**	**30.4 (12.4 – 63.1)**

^a^standardized uptake value.

^b^metabolically active volume as determined from [^18^F]FDG PET.

^c^fractional hypoxic volume.

^d^maximum tumor-to-plasma radioactivity at 90 – 120 min.

**Table 3:** **Biomarker findings from individual patients**

Patient	Ki-67	Glut-1	p53	Hif-1α	CD68	CD31	Tunel	VEGF _SC-152_
no.	%	%	%	%	%	%	%	Intensity
1	17	5	70	0	27	2.5	19	3
2	36	30	90	52	5	0.4	6	2
3	82	10	80	16	26	0.6	10	2
4	18	5	40	10	16	1.8	17	1
5	60	5	95	0	13	5.7	0.3	2
6	35	20	75	0	14	6.0	11	1
7	85	40	25	58	7	6.8	10	2
8	86	60	80	41	30	6.2	7	1
9	87	60	5	33	33	1.6	6	3
10	28	0	75	1	30	2.6	n.d	1
11	80	0	90	1	38	4.7	7	2
12	n.d	n.d	65	68	44	2.6	16	3
13	n.d	50	30	34	27	3.0	20	1
14	35	50	2	23	9	14.9	10	2
15	40	60	2	1	37	7.3	15	1
**Median**	**40**	**25**	**70**	**16**	**27**	**3**	**10**	
**Range**	**17-87**	**0-60**	**2-95**	**0-68**	**5-44**	**0.4-14.9**	**0.3-20**	

n.d. = not determined.

**Table 4:** **Correlations between endogenous biomarkers and PET parameters analyzed from patients with HNSCC**

	[ ^18^F]FDG				[ ^18^F]FETNIM				[ ^15^O]H _2_0	
	SUV ^a^		Tumor volume ^b^ (cm ^3^)		FHV ^c^ (%)		T/P ratio ^d^		Blood flow (ml/100 mg/min)	
	rs	p	rs	p	rs	p	rs	p	rs	p
**GLUT-1**	- 0.166	0.577	- 0.064	0.831	0.333	0.251	- 0.084	0.779	- 0.042	0.895
**HIF-1α**	- 0.194	0.496	- 0.281	0.318	- 0.151	0.598	- 0.345	0.212	- 0.237	0.383
**Ki-67**	0.084	0.781	0.172	0.565	0	1	- 0.046	0.878	- 0.327	0.282
**p53**	0.470	0.078	0.428	0.113	- 0.269	0.340	0.074	0.799	0.020	0.948
**CD-31**	- 0.114	0.691	0.139	0.627	0.314	0.260	0.164	0.566	- 0.360	0.158
**CD-68**	- 0.046	0.872	- 0.007	0.980	0.039	0.892	- 0.093	0.747	- 0.086	0.776
**Tunel**	- 0.414	0.127	- 0.386	0.159	- 0.064	0.824	- 0.125	0.663	0.072	0.810

^a^standardized uptake value.

^b^metabolically active volume as determined from [^18^F]FDG PET.

^c^fractional hypoxic volume.

^d^maximum tumor-to-plasma radioactivity at 90 – 120 min.

r_s_ = Spearman correlation coefficients.

p = p-value.

**Figure 1:**
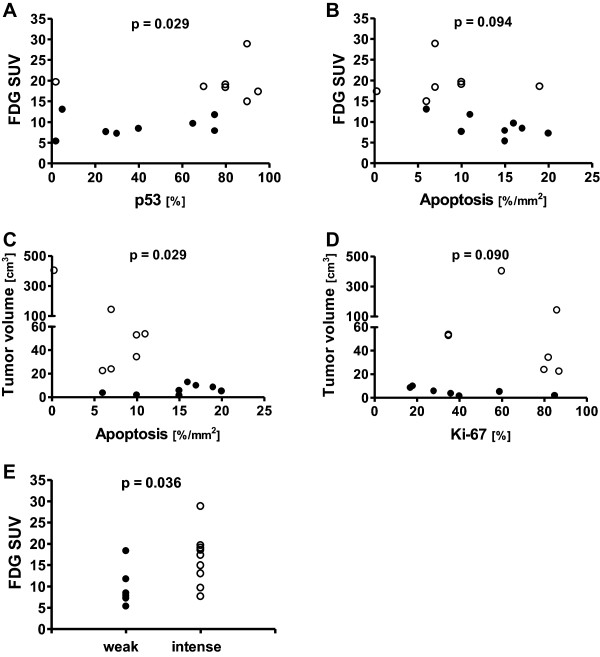
**Relationship between PET findings and immunohistochemistry.** Association between the expression of p53 **(A)** and the amount of apoptotic cells **(B)** with [^18^F]FDG SUV (●, lower SUV; ○, higher SUV). The amount of apoptotic cells displayed an association with the metabolically active tumor volume **(C)** and a trend toward an association in the expression of Ki-67 was also seen with the metabolically active tumor volume **(D)** (●, values less than or equal to the median value; ○, values greater than the median value). In **E**, the association between VEGF expression and [^18^F]FDG SUV is illustrated as ●, weak expression; ○, intense expression.

The PET uptake indices were further divided into values either less than or equal to the median value (Table 2) or values greater than the median value and associated with the expression of biomarkers. As shown in Figure 1A, the tendency toward an association between the expression of p53 and [^18^F]FDG SUV now became statistically significant (p =0.029). There was also a trend that the amount of apoptotic cells would be associated (p =0.094) with [^18^F]FDG SUV (Figure 1B). The metabolically active tumor volume, on the other hand, was inversely associated (p =0.029) with the numbers of apoptotic cells (Figure 1C). The expression of the proliferative marker Ki-67 also showed a clear tendency toward an association (p =0.090) with tumor volume values less than or equal to the median and values greater than the median value (Figure 1D). The expression of VEGF in tumors was analyzed by staining intensity and a significant association was observed with [^18^F]FDG SUV (p =0.036), but not with the other PET parameters (Figure 1E).

### Correlations of biomarker expression

**Figure 2:**
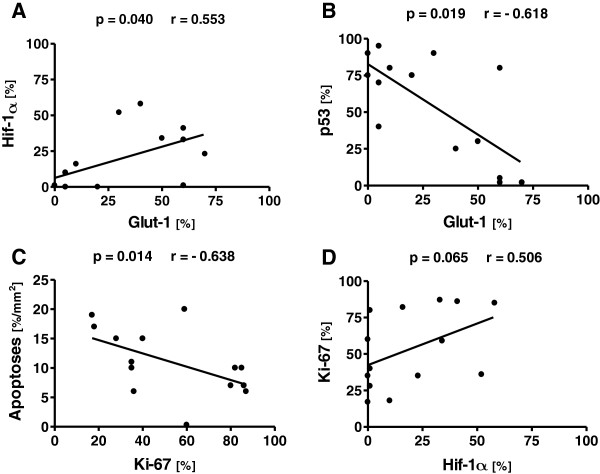
**Correlations of biomarker expression.** A correlation was detected between the expressions of HIF-1α and GLUT-1 **(A)**. The expression of p53 showed a negative correlation with the expression of GLUT-1 **(B)** and the amount of apoptosis with Ki-67 **(C)**. Furthermore, the expression of Ki-67 displayed a borderline correlation with that of HIF-1α **(D)**.

There were significant correlations detected between the expressions of some of the biomarkers. As shown in Figure 2A, the expression of HIF-1α correlated with the expression of GLUT-1 (r =0.553, p =0.040). The expression of p53 correlated inversely (r = −0.618, p =0.019) with the expression of GLUT-1 (Figure 2B). There was also a negative correlation (r = −0.638, p =0.014) between proliferation assessed with Ki-67 and the numbers of apoptotic cells (Figure 2C). There was a trend toward a significant correlation (r =0.506, p =0.065) between the expressions of Ki-67 and HIF-1α (Figure 2D).

### Association between biomarkers and patient outcome

**Figure 3:**
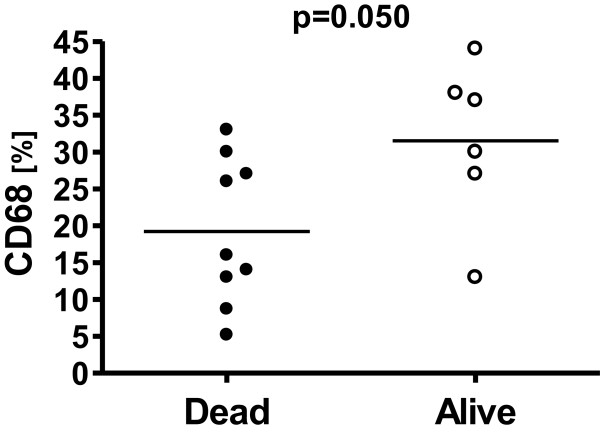
**Association between CD68 and patient outcome.** There was a relationship observed between survival status of patients and the expression of CD68 HNSCC, indicating that an increased rate of macrophage infiltration into tumors was associated with a poor prognosis in patients with HNSCC.

Patient outcome data was available for all patients. At the end of follow-up period, 6 patients were alive and 9 had died of cancer. Of all analyzed biomarkers, only CD68 was associated with overall survival (p =0.050) (Figure 3).

## Discussion

The rational application of hypoxia imaging and hypoxia-directed treatment strategies in oncology has to be based on a fundamental understanding of the biochemical and molecular biological processes that govern the uptake of a given PET tracer. Our study design enabled us to investigate the relationship between protein markers and the uptake of tracers used to image hypoxia, glucose metabolism and blood flow in patients with HNSCC.

### [^18^F]FETNIM uptake, blood flow and biomarker expression

One of our main finding in this study was that the uptake of [^18^F]FETNIM did not correlate with the expression of HIF-1α, nor with any other biomarker analyzed. Similarly, no correlation was seen for blood flow, as assessed using [^15^O]H_2_O, and the expression of the biomarkers depicting the vascular status.

The expression of HIF-1α is elevated in response to hypoxia. HIF-1α induces the expression of hundreds of target genes; those regulating angiogenesis and glucose metabolism are some of the most important with respect to cancer growth [3]. HIF-1α seems to respond not only to hypoxia, but also rapidly to reoxygenation. If this is the case, then HIF-1α may be an unreliable measure of hypoxia in the context of clinical sample collection. Even though several studies have shown a correlation between exogenous markers, such as pimonidazole or EF5, and the uptake of hypoxia radiotracers [19, 20], a poor match between the pimonidazole localization and the distribution of HIF-1α target proteins has been reported [21–23]. Lehmann et al. [21] also failed to find any correlation between HIF-1α expression and [^18^F]FMISO uptake. However, recently two papers reported on a significant correlation between the uptake of [^18^F]FMISO and HIF-1α expression [24, 25] as well as the expression of p53 [25] in HNC. HIF-1α overexpression has also been associated with increased proliferation and p53 expression in invasive breast cancer [26]. In our study, the expression of HIF-1α tended to correlate (r_s_ =0.506, p =0.065) with the expression of Ki-67 (Figure 2D), but not with that of p53. The association between HIF-1α and proliferation is not fully understood - perhaps HIF-1α may either reflect or react to tumor proliferation.

### [^18^F]FDG uptake and GLUT-1 expression

A number of studies have examined the relationship between GLUT-1 expression and the uptake of [^18^F]FDG in head and neck cancer. In the present study, the [^18^F]FDG uptake expressed as SUV did not correlate (r_s_ = -0.166, p =0.577) with the expression of GLUT-1. The lack in correlation between GLUT-1 and [^18^F]FDG SUV has also been reported by others [27–29] in patients with squamous cell carcinoma. On the other hand, other groups have described a positive correlation between [^18^F]FDG SUV and GLUT-1 expression [30–34]. These conflicting findings might, at least partly, depend on the scoring method applied for quantification and hence introduce a systematic bias. This might also be the case for other biomarker analyses performed in this study. In the current study, only cell membrane staining was accounted for regardless of the degree of the cytoplasmic staining pattern or the staining intensity of GLUT-1.

### Relationship between p53, apoptosis, cell proliferation and [^18^F]FDG

Disruption of apoptosis control can lead to unlimited cell growth and promote carcinogenesis. *p53* is one of the most important genes in the regulation of apoptosis. A number of studies have shown that overexpression of mutated p53 protein is associated with poor overall survival in patients with HNSCC [35]. We found a significant association (p =0.029) between p53 expression and the uptake of [^18^F]FDG expressed as SUV (Figure 1A). We also detected a negative association (p =0.029) between the numbers of apoptotic cells and the metabolically active tumor volume (Figure 1C). Studies on epithelial tumors have indicated that tumors with higher apoptotic rates have better prognoses than those with lower rates [36]. Our results revealed higher numbers of cells in apoptosis in smaller tumors (Figure 1C), which furthermore showed a trend toward an association (p =0.094) with a lower [^18^F]FDG SUV (Figure 1B). In line with these findings, higher amounts of apoptotic cells correlated (r_s_ = −0.638, p =0.014) with a lower expression of the proliferative marker Ki-67 (Figure 2C). In summary, our results indicate that tumors with a higher apoptotic rate and reduced p53 expression are less aggressive.

Studies in esophageal cancer [37] and HNSCC [38] with Ki-67 reported that there was a correlation between [^18^F]FDG SUV and Ki-67 but there have also been contradictory studies where no correlation of proliferation with [^18^F]FDG SUV has been observed [39, 40]. In our study, there was no correlation between these two parameters. However, a tendency toward a higher expression of Ki-67 in larger tumors was seen as measured from the metabolically active tumor volume with [^18^F]FDG, but the relationship did not reach statistical significance (p =0.090). Thus, current evidence indicates that while proliferation may contribute to glucose metabolism it is not strongly linked to an increased uptake of [^18^F]FDG and, thus, not glucose.

### Relationship between VEGF and [^18^F]FDG

The expression of VEGF may also stimulate the uptake of [^18^F]FDG in endothelial cells in vitro [41], but this claim has been criticized [26]. Immunohistochemical staining of the VEGF receptor correlates significantly with the uptake of both [^18^F]FDG and [^18^F]FMISO in brain tumors [42]. In a study conducted in esophageal squamous cell cancer patients, the SUV_max_ correlated with the VEGF expression level [43], whereas no such correlation was found in the studies of Taylor et al. [44] and Westerterp et al. [45]. In the current work, there was a positive association (p =0.036) between the staining intensity of VEGF and [^18^F]FDG SUV (Figure 1E), but not with [^18^F]FETNIM.

### Relationship between biological markers and outcome

Only the expression of macrophages (as measured by CD68 staining) was associated with patient outcome. The amount of CD68 positive cells was higher among the surviving patients than among those who died of HNSCC (p =0.050). This finding is in support of the results of a study where the primary tumor macrophage content was a strong predictor of tumor aggressiveness in HNSCC [46]. Although the uptake of [^18^F]FDG could reflect the macrophage content in tumor tissue, we were not able to detect any relationship between these two parameters.

One limitation of the current study, or any study that attempts to correlate PET findings with the expression of biomarkers, is the comparison of findings on a microscopic level (micrometer range) with the PET signal where the resolution is in millimeter range. Furthermore, it is questionable whether the uptake of [^18^F]FDG or [^18^F]FETNIM can reflect only one molecular or cellular rate-limiting step, as e.g. the expression of Glut-1 and HIF-1α.

## Conclusions

A high uptake of [^18^F]FDG expressed as SUV is linked to an aggressive HNSCC phenotype: the rate of apoptosis is low and the expression of p53 and VEGF is high. None of the studied biomarkers correlated with perfusion and hypoxia evaluated with [^15^O]H_2_O-PET and [^18^F]FETNIM-PET, respectively. Estimates of the biomarkers showed that Ki-67 expression was inversely associated with the apoptotic rate, which further supports the concept that the apoptotic rate reflects the prognosis.

In conclusion, [^18^F]FDG uptake is associated with the expression of p53 and with apoptosis in HNSCC. Still, the overall uptake of a tracer into HNSCC is clearly the net sum of multiple mechanisms, since no other associations were detected, e.g., there were no statistically significant correlations found between [^18^F]FETNIM-PET and HIF-1α, [^15^O]H_2_O-PET and microvessel density. Research in this area is warranted to clarify the molecular pathways underlying tracer uptake.

## Abbreviations

CD68: Macrophage antibody
CD31: Endothelial cell antibody
CT: Computer tomography
DNA: Deoxyribonucleic acid
[^18^F]FDG: 2-[^18^F]: [^18^F]fluoro-2-deoxy-D-glucose
[^18^F]FETNIM: [^18^F]fluoroerythronitroimidazole
[^18^F]FMISO: [^18^F]fluoromisonidazole
FHV: Fractional hypoxic volume
GBq: Gigabecquerel
Gy: Gray
GLUT-1: Glucose transporter-1
HIF-1α: Hypoxia-inducible transcription factor-1alpha
HE: Hematoxylin and eosin
HNSCC: Head and neck cancer squamous cell carcinoma
HRE: Hypoxia-response element
[^15^O]H_2_O: [^15^O]labeled water
IHC: Immunohistochemistry
i.v.: Intravenous
Ki-67: Proliferation antibody
MBq: Megabecquerel
p53: Tumour suppressor p53
PET: Postron emission tomography
r_s_: Spearman´s correlation coefficient
ROI: Region of interest
RT: Radiotherapy or room temperature
SD: Standard deviation
SUV: Standardized uptake value
T/P: Tumour-to-plasma ratio
T/V: Metabolically active tumour volume
VEGF: Vascular endothelial growth factor.
